# Acid-Catalyzed Conversion of Cellulose Into Levulinic Acid With Biphasic Solvent System

**DOI:** 10.3389/fpls.2021.630807

**Published:** 2021-03-17

**Authors:** Changyue Ma, Bo Cai, Le Zhang, Junfeng Feng, Hui Pan

**Affiliations:** ^1^Jiangsu Provincial Key Lab for the Chemistry and Utilization of Agro-forest Biomass, Nanjing Forestry University, Nanjing, China; ^2^College of Chemical Engineering, Nanjing Forestry University, Nanjing, China

**Keywords:** cellulose, Amberlyst-15, biphasic solvent system, levulinic acid, catalyst

## Abstract

In this work, acid-catalyzed conversion of cellulose into levulinic acid in a biphasic solvent system was developed. Compared to a series of catalysts investigated in this study, the Amberlyst-15 as a more efficient acid catalyst was used in the hydrolysis of cellulose and further dehydration of derived intermediates into levulinic acid. Besides, the mechanism of biphasic solvent system in the conversion of cellulose was studied in detail, and the results showed biphasic solvent system can promote the conversion of cellulose and suppress the polymerization of the by-products (such as lactic acid).The reaction conditions, such as temperature, time, and catalyst loading were changed to investigate the effect on the yield of levulinic acid. The results indicated that an appealing LA yield of 59.24% was achieved at 200°C and 180 min with a 2:1 ratio of Amberlyst-15 catalyst and cellulose in GVL/H_2_O under N_2_ pressure. The influence of different amounts of NaCl addition to this reaction was also investigated. This study provides an economical and environmental-friendly method for the acid-catalyzed conversion of cellulose and high yield of the value-added chemical.

## Introduction

Renewable biomass has attracted widespread attention due to the environmental concern placed on greenhouse gas emission by the intensive consumption of fossil oil and the diminishing fossil resources. Efficient use of abundant biomass cannot only reduce the problem of environment pollution and the heavy dependence on fossil fuels, but also meet the future energy needs and follow the principle of green chemistry (Huber et al., 2006; Rengsirikul et al., 2013). Cellulose is one of the three major components and takes 30–50% mass content of lignocellulosic biomass. It can be converted to platform molecules and high-value chemicals, such as sugar alcohols, 5-hydroxymethylfurfural (HMF), lactic acid, levulinic acid (LA), ethylene glycol, and alkanes (Qi et al., 2011; Weingarten et al., 2012; Mukherjee et al., 2015). Among these products, LA is one of the 12 most important platform chemicals derived from biomass that have been announced by the U.S. Department of Energy, which exhibits excellent stability and has wide applications in organic synthesis, the pharmaceuticals industry, and agriculture (Li et al., 2019). As the typical platform chemical is derived from biomass, LA can be converted to high added-value fuel additives such as γ-valerolactone (Xu et al., 2019; Yi et al., 2020).

**SCHEME 1:**
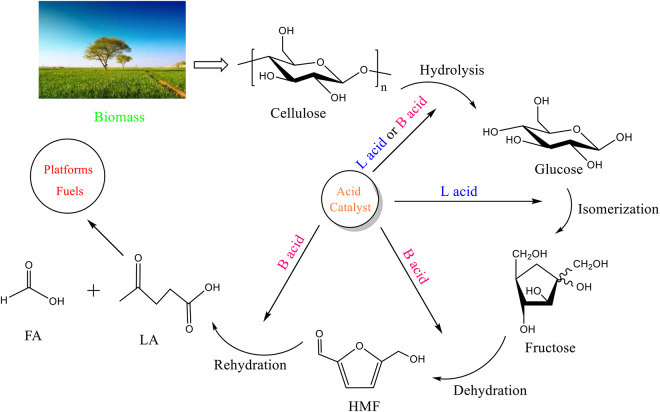
Reaction pathway from cellulose to LA.

The process of preparing levulinic acid from cellulose mainly includes the following four steps: (1) cellulose decomposed to glucose through acid hydrolysis and (2) the isomerization of glucose to fructose in the presence of Lewis acid (L acid) (Tang et al., 2015); (3) Fructose is converted to HMF via dehydration with Brønsted acid (B acid) and (4) consequently rehydrated to LA and formic acid (Scheme 1).

In the commercial production process of levulinic acid, the method using diluted mineral acid to catalyze two coherent steps, gives a satisfactory LA yield based on the theoretic molar yield (Sairanen et al., 2014; Ghosh et al., 2016). However, the usage of inorganic acid has several disadvantages, such as difficulty of recycling, equipment corrosion, and serious environmental problems, which greatly inhibit its widespread applications (Mascal and Nikitin, 2008). Comprehensive efforts have contributed to the development of heterogeneous catalyst for the preparation of LA (Mascal and Nikitin, 2008; Ding et al., 2014). Various Lewis acids have been applied for carbohydrate conversion in recent researches. Zhao et al. studied the effect of different metal chlorides on the conversion of sugars to 5-HMF in ionic liquids (Zhao et al., 2007). The tandem conversion of glucose to 5-HMF in aqueous CrCl_3_-HCl solution was investigated (Dallas Swift et al., 2015). The conversion of glucose to 5-HMF reached about 70% yield, which was catalyzed by chromium(II) chloride (Zhao et al., 2019). Compared with mineral acid, solid acid catalysts are more suitable for the production of LA from cellulose because these catalysts have low corrosiveness and easy recoverability. Previous research reported that hydrolysis of cellulose catalyzed by Nafion SAC-13 and FeCl_3_based on amorphous silica was highly dependent on the temperature of reaction (Hegner et al., 2010). Moreover, the use of SnCl_4_ could achieve 64% yield of 5-HMF from glucose in ionic liquid (Cao X. et al., 2015). However, some ions, such asSn^4+^ are toxic, and ionic liquids are specialty solvents with a high price (Zhang and Zhao, 2009). Therefore, a more environmentally friendly catalyst needs to be found for the production of LA.

In addition to the catalyst, the optimization of the reaction medium for LA production is another main strategy that current research activities have focused upon. The use of appropriate solvents can enhance the solubility of cellulose, which increases the rates of mass transfer between the biomass and the catalysts, and consequently enhances the apparent reaction rates and conversion. Some studies have shown interest in biphasic reaction mediums that contain water and a polar aprotic solvent (e.g., γ-valerolactone (GVL), tetrahydrofuran (THF), dimethyl sulfoxide (DMSO), and sulfolane).These reaction mediums could take advantage of the differences in hydrophobicity of products and reactants, which may usually lead to a higher yield of target product than that with monophasic or aqueous systems. Furthermore, the application of polar aprotic solvent such as DMSO and GVL shows beneficial effects on the destabilization of acidic proton and suppression of glucose degradation (Chen et al., 2017). Meanwhile, a minimum amount of water in aprotic solvent can promote the dissolution of biomass-derived materials, while the aprotic solvent can improve reaction performance. For instance, 17 mol% LA produced by using Amberlyst-36 efficiently from vegetable waste in DMSO-water mixture (Chen et al., 2017). Dumesic et al. used GVL/water as solvent and H_2_SO_4_ as an acid-catalyst for extracting lignin from lignocellulosic biomass, which indicated that GVL facilitates complete solubilization of the biomass (Luterbacher et al., 2014). Moreau et al. (2006) reported that up to 92 mol% of 5-HMF yield could be achieved from fructose using 1-H-3-methylimidazolium chloride as reaction medium and Amberlyst-15 as catalyst in 45 min. Choudhary et al. (2013) achieved 59% yield of 5-HMF and 46% yield of LA from glucose in water/THF biphasic solvent systemwithCrCl_3_ and HCl as catalysts. The microwave-assisted hydrolysis of bamboo to 5-HMF and furfural is also studied in a dilute acid (H_2_O)/methyl isobutyl ketone (MIBK)biphasic system (Sweygers et al., 2020). Generally, in a biphasic system, the hydrolysis of cellulose to glucose and the further degradation of glucose to 5-HMF occur mainly in the aqueous phase, and the degradation of 5-HMF to LA mainly occurs in the organic phase (Ghosh et al., 2016; Lang et al., 2020). This separated reaction medium offers many advantages such as enhancing cellulose solubility, preventing LA from polymerization and concentrating products by using a lower volume of solvent. Therefore, using biphasic solvent systems that contain water and polar aprotic solvent could promote the intermediates or product transfer from the aqueous phase to the organic phase, make the reaction forward, and improve the selectivity and yield of LA, which has great research significance.

In this work, different catalysts, including homogeneous Lewis acid and heterogeneous Brønsted acid were employed to catalyze the conversion of cellulose to LA and their catalytic activities were compared. The influence of different biphasic solvent systems that consists of water and a polar aprotic solvent, including GVL, THF, 1,4-dioxane (DIO), sulfolane and DMSO, and their role in the hydrolysis of cellulose was also investigated. Furthermore, the effects of reaction conditions such as reaction temperature and time, the loading of catalysts, N_2_ pressure and NaCl dosage on cellulose conversion and yield of LA were also explored. We demonstrated a preferable method to easily produce LA with high yield from cellulose under N_2_ pressure in H_2_O/GVL biphasic solvent system with Amberlyst-15. Overall, this one-pot directional catalytic strategy is a high-efficiency and eco-friendly route for conversion of cellulose to high-value chemicals.

## Experimental

### Materials

Microcrystalline cellulose (96%, 20 μm, Sigma-Aldrich), glucose (98%), oxalic acid dihydrate, Amberlyst-15 and all polar aprotic solvents (e.g., GVL, DMSO, THF, DIO, and sulfolane) were supplied by TCI Chemicals Co. Ltd (Shanghai, China). 5-Hydroxymethylfurfural (HMF, 99%) and fructose were purchased from Adamas-beta Inc. (Shanghai, China). HZSM-5 (SiO_2_/Al_2_O_3_ = 25) was obtained from Nankai University Catalyst Co., Ltd. (Tianjin, China). Other catalysts such as metal salts were purchased from Sinopharm Chemical Reagent Co., Ltd (Shanghai, China). All chemicals were of analytical grade and directly used without any further pre-treatment.

### Catalytic Conversion of Cellulose

All reactions were carried out in an autoclave with a total volume of 30 mL. A typical run was performed as follows: Firstly, microcrystalline cellulose, solvent, and catalyst were added into the reactor. The reactor was then sealed and heated. After cooling to room temperature, the reaction mixture was pumped to separate the solid from the solution. The liquid part was filtered with a 0.22 mm membrane filter before HPLC analysis, while the solid residues were washed with deionized water for three times to remove soluble products and remaining solvent. The catalyst Amberlyst-15 and humins were separated from the washed solid residues by a 60-mesh sieve and then dried overnight at 105°C for further microscopic and spectroscopic characterizations.

The conversion of the cellulose was calculated from the equation:

$$\mathrm{Cellulose}\,\mathrm{conversion}={\frac{\mathrm{weight}\,\mathrm{of}\,\mathrm{cellulose}\,\mathrm{reacted}}{\mathrm{weight}\,\mathrm{of}\,\mathrm{initial}\,\mathrm{cellulose}}}^{*}\,100\%$$

The yields and selectivity of products were determined by the following formulas:

$$\mathrm{Products}\,\mathrm{yield}={\frac{\mathrm{weight}\,\mathrm{of}\,\mathrm{glucose},\mathrm{fructose},5-\mathrm{HMF},\mathrm{LA}\,\mathrm{and}\,\mathrm{FA}}{\mathrm{weight}\,\mathrm{of}\,\mathrm{initial}\,\mathrm{cellulose}}}^{*}\,100\%$$

$$P\,r\,o\,d\,u\,c\,t\,s\,s\,e\,l\,e\,c\,t\,i\,v\,i\,t\,y={\frac{\mathrm{products}\,\mathrm{yield}}{\mathrm{cellulose}\,\mathrm{conversion}}}^{*}\,100\%$$

### Product Analysis

The reaction intermediates and products including glucose, fructose, 5-HMF and levulinic acid were analyzed on an Agilent 1200 series HPLC using a Bio-Rad AminexHPX-87H column (300 m × 7.8 mm) operating at 55°C with a refractive index (RI) detector. H_2_SO_4_ aqueous solution (5 mM) was used as the mobile phase with a flow rate of 0.6 mL/min and the injection volume of the sample was 5 μL.

The FT-IR analysis of the reaction residues was performed on an IS-10 Fourier transform infrared spectrometer from Nicolet Company (America). To prepare solid testing samples, the residual powder and potassium bromide powder were mixed at a certain mass ratio and ground in a mortar. Mixed powder was then pressed into a sample tablet with a thickness of about 1 mm. The scanning range was 4,000∼400 cm^–1^ with a resolution of 4 cm^–1^. The scanning signal was accumulated 16 times, and the interference of water and carbon dioxide was deducted during the scanning.

Powder X-ray diffraction patterns (XRD) were gained with a Rigaku powder X-ray diffractometer using Cu Kα radiation (λ = 0.1542 nm). The scan range is from 5 to 45°. Nitrogen physisorption was conducted at -196°C on a Micromeritics ASAP 2020 M apparatus.

## Results and Discussion

### Catalyst Screening

The conversion from cellulose to LA needs both Lewis acid for the isomerization of glucose to fructose and Brønsted acid for dehydration of fructose to HMF and rehydration of HMF to LA (Tang et al., 2015; Huang et al., 2018). The catalysts with Lewis acid or/and Brønsted acid sites were selected in this study for the cellulose conversion. Moreover, it has been reported that excess catalysts would lead to the generation of humins and reduce the yield of LA (Ji et al., 2019). Therefore, the amounts of the catalysts were firstly optimized (Supplementary Tables S1–S3) and Table 1 lists the cellulose conversion, yield and selectivity of LA using selected catalysts with their optimized dosages. HCl is a strong mineral acid and could convert cellulose to LA with high yield as expected. However, it could corrode equipment and is difficult to be recycled and reused owing to its high-water miscibility. And so does the purification of reaction products in the homogeneous reaction medium. Oxalic acid only gave 1.57l% yield of LA due to its weak acidity. Preview literature studied that when the sulfonic acid group (SO_3_-H) was successfully grafted onto zeolite structure, maximum LA yield of 31.15% was obtained with 3% S-βcatalyst using fructose as material (Bisen et al., 2020). Amberlyst-15 could obtain a LA yield of 29.91% and cellulose conversion of 71.29%. Although its yield of LA is slightly lower than that of FeCl_3_, Amberlyst-15 has higher selectivity of target product LA. In addition, Amberlyst-15 is a heterogeneous Brønsted acid catalyst, which offers an environmental advantage because it can be easily separated and recycled compared to other tested homogenous metal salt catalysts. Therefore, Amberlyst-15 was selected in this study for further investigation.

**TABLE 1:** Effect of different catalysts on cellulose conversion and LA yield.

Entry	Catalyst	LA/%	Conversion/%	Selectivity of LA/%
1^a^	HCl	50.81	94.7	53.65
2^a^	Oxalic acid	1.57	37.22	4.22
3^b^	Amberlyst-15	29.91	71.29	41.96

*^*a*^Reaction condition: cellulose 100 mg, catalyst 100 mg, H_2_O 12 mL, 180°C, 180 min. ^*b*^Cellulose 100 mg, catalyst 300 mg.*

### Effect of Different Solvent Systems

To further evaluate the effect of reaction medium on the cellulose conversion to LA, different solvent systems were tested and the results are shown in Table 2 and Supplementary Table S6. The yields of intermediate products were very low and the main product is the target products, LA. In general, the cellulose conversion and yield of LA were all improved when an aprotic solvent was introduced to pure water system except for sulfolane and DMSO. Biphasic reaction medium is believed to have many advantages for biomass hydrolysis as reported in literature: (1) target products such as LA and HMF were extracted to the organic layer during the reaction, which could prevent degradation or polymerization in the aqueous layer (Wang et al., 2012); (2) the intermediate product glucose in the aqueous phase can be continuously converted to LA as the target products were extracted to organic phase (Mellmer et al., 2019); (3) a water-rich local solvent domain could be formed around the hydroxyl group in reactant (cellulose) and main intermediates (glucose, 5-hydroxymethylfurfural) in biphasic solvent systems, where reactant and main intermediates can be easily rehydrated and therefore promote the formation of LA (He et al., 2017). Therefore, using biphasic systems could lead to less solid residues after reaction and potentially accelerate the rate of reactions (Yu et al., 2017).

**TABLE 2:** Effect of different biphasic solvent systems on cellulose conversion and LA yield^a^.

Entry	Solvent	Temp./°C	LA/%	Conversion/%	Selectivity of LA/%
1	H_2_O	180	29.91	71.29	41.96
2	GVL/H_2_O	180	36.90	93.83	39.33
3	GVL/H_2_O	200	50.40	83.54	60.33
4	THF/H_2_O	180	47.73	94.25	50.64
5	THF/H_2_O	200	34.80	87.16	39.93
6	DIO/H_2_O	180	32.18	81.04	39.71
7	Sulfolane/H_2_O	180	22.58	83.71	26.97
8	DMSO/H_2_O	180	5.69	42.33	13.44

*^*a*^Reaction condition: cellulose 100 mg, Amberlyst-15 300 mg, 180 min, solvent 12 mL, ratio of biphasic solvents: 1:1.*

In the DMSO/H_2_O biphasic solvent system, the yield of LA is only 5.69% and the conversion is 42.33%. Similarly, the yield of LA and conversion of cellulose in the sulfolane/H_2_O biphasic solvent system were 22.58 and 83.71%, respectively. Accounting for DMSO has a higher polarity and dipole moment in comparison with the other solvents (Cao et al., 2015). Meanwhile, although DMSO and sulfolane, as polar aprotic solvents with high boiling points (Supplementary Table S5), contribute to the isomerization of glucose to fructose, DMSO especially has catalytic effect on the dehydration of fructose to HMF, but does not have much effect on the hydrolysis of cellulose to LA (Sim et al., 2012; Chen et al., 2017). Therefore, the lower yield of LA happened in the DMSO/H_2_O and sulfolane/H_2_O though they do play a role in the formation of biphasic solvent systems.

The THF/H_2_O and GVL/H_2_O reaction mediums demonstrated relatively better performance among these biphasic solvent systems in terms of the yield of LA and conversion of cellulose. THF could protect and transfer the cellulose-derived products to organic phase by forming hydrogen bonding between oxygen atom in THF and hydrogen atom of C4-O-H in glucose or aldehyde group in 5-HMF, which can increase the yield of LA (Jiang et al., 2018). However, THF is toxic and may cause environmental pollution, while GVL is a green and environmentally friendly solvent. One particular advantage of GVL/H_2_O system is that lower acid concentrations were used to produce LA compared to pure water as the solvent. Additionally, GVL/H_2_O solubilized both cellulose and humins to prevent solid accumulation in the reactor, which could help implement continuous flow reactors and eliminate the filtration of solids (Alonso et al., 2013). Besides, the hydrolysis reaction is 100 times faster in GVL than in water (Mellmer et al., 2014). Increasing reaction temperature from 180 to 200°C could further promote the yield of LA from 36.90 to 50.40% (entry 3) in GVL/H_2_O and from 47.73 to 34.80% in THF/H_2_O (entry 5). Furthermore, the conversion of cellulose was decreased both in THF/H2O and GVL/H_2_O system, which may result in higher temperatures, causing the formation of humin. Meanwhile, selectivity of LA in GVL/H_2_O (60.33%) is higher than that in THF/H_2_O (39.93%) under 200°C. Hence, the GVL/H_2_O biphasic solvent system was chosen for the following optimization experiment.

### Effect of Other Experimental Parameters

**FIGURE 1:**
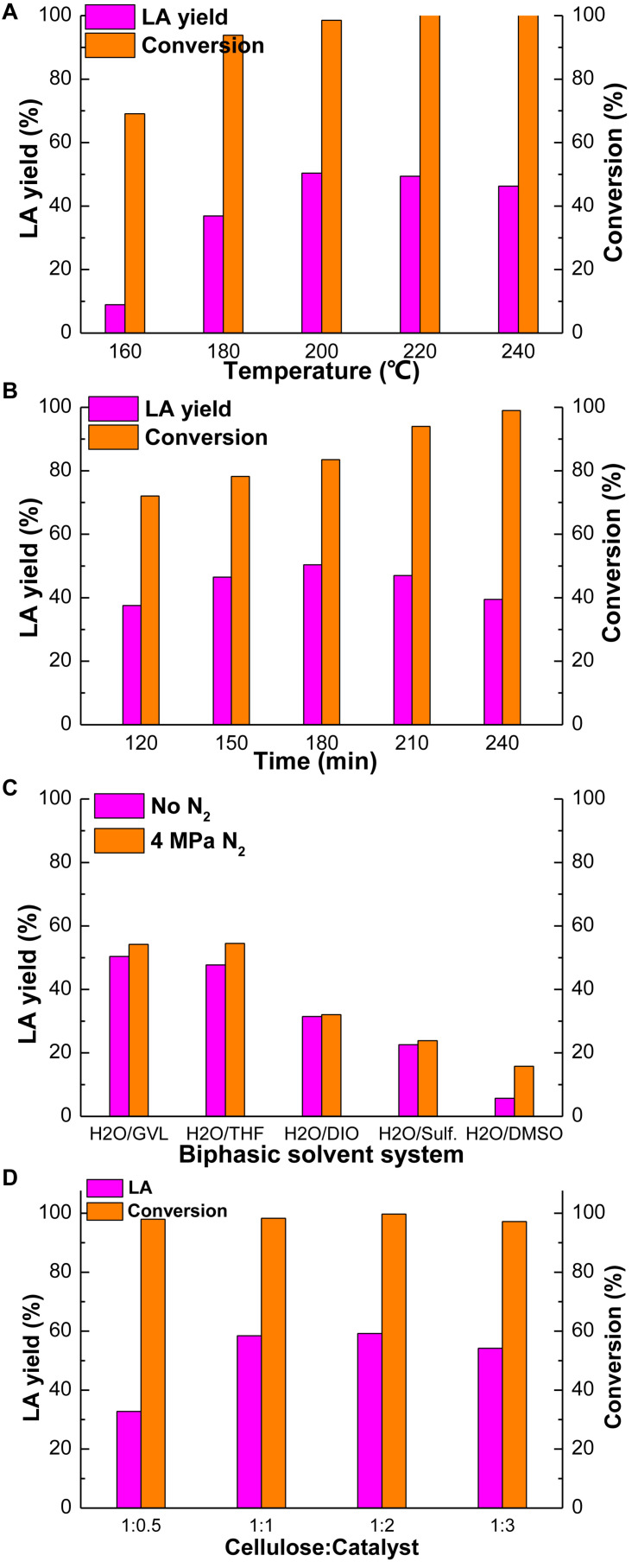
Effect of reaction temperature **(A)**, reaction time **(B)** in GVL/H_2_O, N_2_ pressure **(C)** and catalyst loading**(D)** on cellulose conversion. Reaction condition: **(A)** cellulose 100 mg, Amberlyst-15 300 mg, H_2_O 6 mL, GVL 6 mL, 180 min; **(B)** cellulose 100 mg, Amberlyst-15 300 mg, H_2_O 6 mL, GVL 6 mL, 200°C; **(C)** cellulose 100 mg, Amberlyst-15 300 mg, 180 min, 200°C, ratio of biphasic solvent: 1:1, 4 MPaN_2_; **(D)** cellulose 100 mg, H_2_O 6 mL, GVL 6 mL, 180 min, 200°C, 4 MPaN_2_.

The effect of other reaction conditions was investigated and the figures were shown in Supplementary Tables S7–S10. The yields of intermediate products were very low under above reaction conditions and the main product is the target products, LA. Except at the temperature of 160°C, the yield of glucose is obvious (28.32%), because this is the suitable temperature for glucose production. In general, a proper reaction temperature is the key to prevent most side-reactions and therefore the yields of desirable products (Yan et al., 2008). The influence of reaction temperature on cellulose conversion in GVL/H_2_O biphasic solvent system is shown in Figure 1A. The LA yield increased significantly from 8.94 to 50.40% as the reaction temperature increased from 160 to 200°C. Then it gradually decreased when the reaction temperature further increased. The yield of LA reduced dramatically to 46.31% as the reaction temperature reached 240°C. This result should be due to the reason that LA might decompose to by-products at higher temperatures (Weingarten et al., 2012).

Similarly, reaction time can also affect the yield of products and the conversion of cellulose. Figure 1B demonstrates the influence of reaction time on cellulose conversion and the production of LA in GVL/H_2_O. As the reaction time prolonged from 120 to 180 min, the conversion of cellulose and the yield of LA increased from 72.04 and 37.56% to 83.54 and 50.40%, respectively. Then the yield of LA began to gradually decrease as the reaction time further increased beyond 180 min. With the reaction time of 240 min, the yield of LA declined to 39.48% and the conversion of cellulose rose to 99.01%. Excessive reaction time may lead to polymerization of the products and result in lower yield of target products. Moreover, humins may be formed from the cellulose if the reaction time is too long (Yan et al., 2008).

The effect of N_2_ pressure on cellulose conversion in GVL/H_2_O was investigated with the range from 0 to 5 MPa (Supplementary Figure S1) and 4 MPa was the best level of pressure, under which the LA yield was 54.21% and the cellulose conversion was 97.17%. It is obvious that with the increasing of N_2_ pressure, the yield of LA and the conversion of cellulose have risen up, which rose slightly (54.51 and 99.9%) when it reached 5 MPa, however. Considering the cost of the experiment and the production yield, 4 MPa N_2_ pressure was the best condition of pressure. Figure 1C shows the influence of N_2_ pressure in different biphasic solvent systems on cellulose conversion. It can be seen that with the introducing of 4 MPaN_2_ pressure, the yields of LA increased obviously in all three investigated reaction mediums of GVL/H_2_O, THF/H_2_O, and DMSO/H_2_O. In addition, *in situ* pressure changes of both reaction systems w/o N_2_ pressure were monitored during the reaction. It showed that, without the introducing of N_2_, around 1 MPa pressure was generated due to the vaporization of the solvents during the reaction process. When adding 4 MPaN_2_ to the autoclave, the maximum pressure in the reactor could reach 7 MPa during the reaction. Additional N_2_ pressure could contribute to the improved conversion of cellulose and the yield of LA by limiting the reactant to a local area, therefore increasing the association between the reactant and protons, and also stabilizing the carbon transition state in the acid-catalyzed reaction (Duan et al., 2019).

Figure 1D displays the influence of catalyst loading on cellulose conversion and the yield of LA, which can be seen that when the ratio of cellulose and catalyst was 1:2, the yield of LA and conversion of cellulose reached the maximum, 59.24 and 99.70%, respectively. Then they began to slightly decrease as catalyst loading further increased to 1:3, which were 54.21 and 97.17%, respectively. This may because of the excess Lewis acid sites which would lead to more side-reactions such as formation of more humins (Zhou et al., 2019).

Overall, the optimum reaction condition is 100 mg cellulose and 200 mg Amberlyst-15 in 12 ml GVL/H_2_O (1:1) at 200°C, 180 min, 4 MPaN_2_, under which the maximum yield of LA is 59.24% and the conversion of cellulose is 99.70%.

Previous research reported that NaCl could promote the depolymerization of cellulose and improves the generation of acidic products by pushing protons to the surface of cellulose and increasing surface acidity (Potvin et al., 2011; Jiang et al., 2019). The effect of different dosage of NaCl on cellulose hydrolysis was explored and the results are shown in Supplementary Figure S3. However, in this reaction, the addition of NaCl did not improve the yield of LA and conversion of cellulose. This may be attributed to the fact that NaCl could help the hydrogen atoms of the hydroxyl group on C_1_ and C_6_ in glucose form hydrogen bonds and promote the dehydration of glucose to form LA. Excessive ion dispersion in the reaction solvent reduces the chance of contact of the protons in the solvent with the reactants, thereby reducing the reactivity of the catalyst. What is more, salt can lead to corrosion of the reactor and create an additional waste stream, leading to an unsustainable process (Sener et al., 2018).

The recycling of Amberlyst-15 was evaluated and shown in Supplementary Figure S4. It can be seen that both the cellulose conversion and the LA yield obviously decreased from 97.17 to 82.78% and from 54.21 to 48.21%, respectively, when the catalyst was used twice. It indicated that in the GVL/H_2_O biphasic solvent system, Amberlyst-15 was unrecoverable because of N_2_ pressure, while in other biphasic solvent systems, the catalyst was deactivated after just one reaction.

Some methods of separation and purification of LA from preview literature could be applied in our experiments. A granular activated carbon (GAC) adsorption method for separation of lanthanum and formic acid was studied (Liu et al., 2012). Kim et al. also confirmed that LA could be effectively separated from 5-HMF by ED (Kim et al., 2013). What is more, Habe et al. (2017) found that the application of desalting electrodialysis (ED) to purify LA may be a favorable method for recovering LA from cedar-based LA solutions.

**FIGURE 2:**
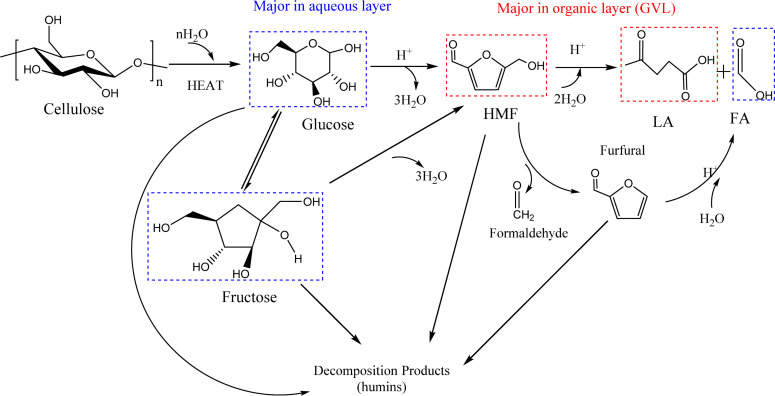
Overall reaction pathway for acid-catalyzed production of LA from cellulose in biphasic solvent systems. The main intermediate compounds and target products are detected by HPLC.

Intermediate compounds were investigated to determine the reaction route. Moreover, owing to verify the reaction process, different substrates (glucose, fructose and 5-HMF), the main intermediate products of cellulose conversion to LA, were used for comparative experiments to detect the distribution of the reaction products (Supplementary Table S5). It is apparent that the yield of LA reached the maximum (52.93%) when 5-HMFwas used as the substrate. When the substrate was glucose, the yield of LA was 31.88%, which was lower than that of fructose (38.92%). And during the reaction of glucose, fructose (2.90%) was generated as the mid-product. Thus, the reaction route has been investigated, which is shown as Figure 2. Firstly, as cellulose has better solvation in GVL than in water (Mellmer et al., 2014), an improved conversion of cellulose in GVL/H_2_O mixture could be attributed to higher solubility of cellulose and faster mass transfer of the hydrolyzed glucose, which is produced by cellulose initially hydrothermally broken down due to the strong interaction between Amberlyst-15 and β-1,4-glycosidic bonds in cellulose. The competition for protons is weaker between glucose and GVL than glucose and water. Hence, the presence of GVL is more conducive to glucose protonation at C2-OH group by increasing the accessibility of its hydroxyl groups to more protons (Qian, 2011; Qian and Liu, 2014). Then there are three parallel pathways for the glucose reaction: (1) decomposition to form humins; (2) fructose the isomerization of glucose is produced by isomerization reaction, and (3) Brønsted acid-catalyzed formation of HMF is initiated by protonation of glucose at O5 position (Yang and Pidko Hensen, 2012). The reversion and epimerization products can also decompose to the formation of humins. Fructose can also dehydrate to form HMF. Subsequently, HMF is rehydrated to form LA and formic acid. Besides glucose polymerization into humins, HMF is also known to form humins under acid catalysis via aldol condensation with an intermediate of 2,5-dioxo-6-hydroxyhexanal, and through the loss of formaldehyde to produce trace furfural (Patil et al., 2012; Tsilomelekis et al., 2016). In contrast, LA is less likely to be transformed to humins and remains steady with the reaction time during acid catalysis. Formic acid is also a by-product of furfural by hydrolysis and fission (van Zandvoort et al., 2015). Yet, in this reaction, the products degraded in the aqueous layer would transfer to the organic layer (GVL), such as HMF and LA, due to the difference in hydrophobicity between the reactants and the products, while the sugars and acids remain in the aqueous layer, which lead to the efficient separation of products and the recovery of solvents (Mellmer et al., 2019; Lang et al., 2020). What is more, the proton transition states of acidic protons in polar aprotic solvents, such as GVL and THF, are unstable relative to water, where destabilization of the acidic protons could lead to the increased reactivity of acid-catalyzed reactions (Wang et al., 2012; Mellmer et al., 2019). The higher selectivity of LA in GVL/H_2_O illustrates the solvent effect. In GVL/H_2_O, the hydrogen bonding between the Brønsted acid site on Amberlyst-15 and GVL (polar aprotic solvent) is not as strong as that in aqueous medium. Furthermore, in the GVL/H_2_O mixture, the rehydration rate of HMF to LA is faster because GVL dissolves HMF preferably over water, which can maximize the degradation of HMF by promoting nucleophilic attack at its carbonyl group (Tsilomelekis et al., 2016). In addition, GVL lowers not only the activation energy for glucose dehydration via C2-OH protonation, but also the activation energy for humins formation via C1-OH protonation. Therefore, the formation of humins remains a major hurdle in GVL/H_2_O, which is more significant than that in aqueous medium.

### Products Characterization

**FIGURE 3:**
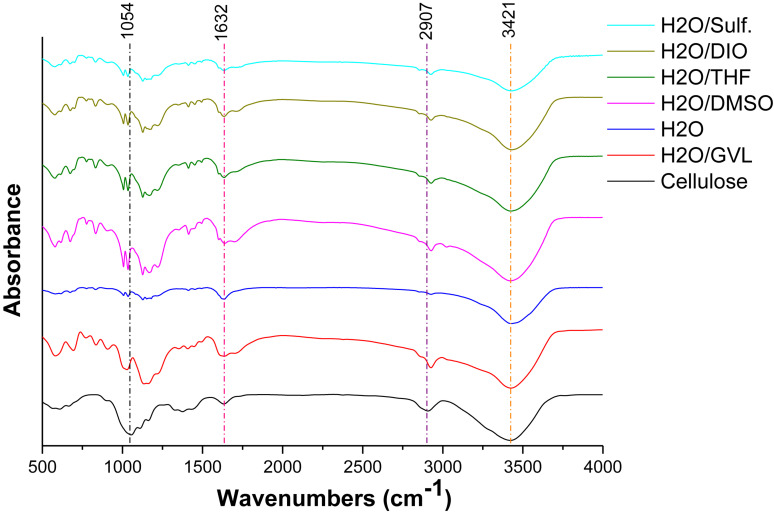
FTIR spectra of cellulose and solid residues after reactions with different biphasic solvent systems.

Figure 3 displays the FTIR spectra of original cellulose and the solid residues from the reactions with different solvent systems. For a typical FTIR spectrum of cellulose, the absorption peaks at 3,421 and 1,316 cm^–1^ correspond to the stretching and bending of hydroxyl groups, respectively; the peak at 2,907 cm^–1^ is ascribed to C-H stretching; the absorption at 1,632 cm^–1^ is attributed to the bond of water in samples (Zhao et al., 2018). It is notable that all solid residues from different reaction mediums exhibit similar FTIR spectra as that of original cellulose. The above absorption peaks could also indicate the fundamental framework of polysaccharides remained in the humins (Zhao et al., 2018). This fact might be the result of insufficient hydrolyzation of cellulose, as well as the further condensation of the degraded intermediates (Wang et al., 2012). Hence, it is speculated that the residue is humins including cellulose which has not been hydrolyzed and polymer of the intermediates.

**FIGURE 4:**
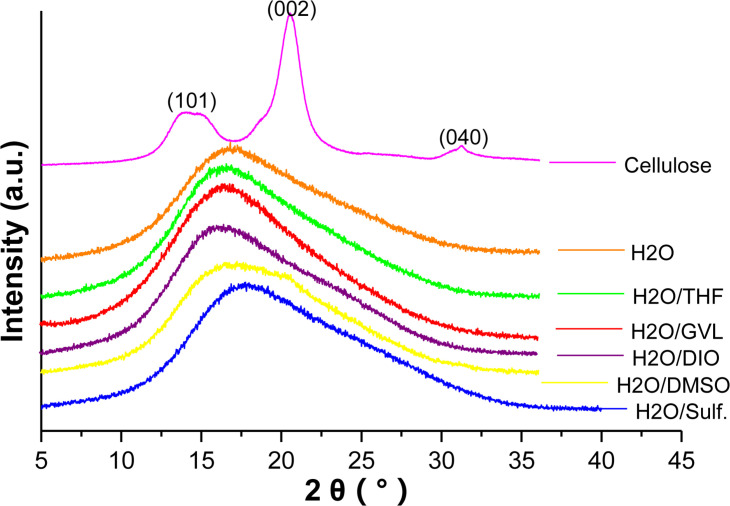
X-ray diffraction patterns of cellulose and solid residues after reactions with different biphasic solvent systems.

Figure 4 shows the X-ray diffraction patterns of cellulose and the solid residues from different reaction systems. For the original cellulose, three main peaks located at 15.5, 22.4, and 34.4° assigned to the (101), (002), and (040) planes, respectively, of cellulose I can be clearly identified (Xie et al., 2016). Nevertheless, none of the XRD pattern of the solid residue displays such peaks of cellulose I. Instead, all of the solid residues from different solvent systems show similar amorphous structures, indicating the loss of crystallinity of cellulose after the reaction. Previous literature claim that hydrolysis of cellulose could result in the broadening and shifting of the characteristic peaks of its crystal structure (Weingarten et al., 2012). What is more, crystallinity of cellulose could be destructed under the high temperature such as this reaction. Therefore, it is further confirmed that residue is humins formed bypure cellulose that lost crystallinity and polymerization of the intermediates, which is consistent with the result of FTIR.

## Conclusion

In summary, Amberlyst-15 identified as an efficient catalyst and GVL/H_2_O as a biphasic solvent system was investigated in the acid-catalyzed conversion of cellulose into LA, where 59.24% yield of LA was achieved at 200°C, 180 min under 4 MPa N_2_ pressure. During the reaction process, LA products that might degrade from the aqueous layer division to the organic layer while the intermediate product (such as sugars) and acids remain in the aqueous layer. This could promote the production of levulinic acid, help the purification and isolation of the product as well. Therefore, the conversion of cellulose and the yield of LA in the biphasic solvent system such as GVL/H_2_O (99.70 and 59.24%, respectively) are higher than those in the pure water system (71.29 and 29.91%). NaCl also has some impact on the conversion of cellulose, which promotes the depolymerization of cellulose and enhances the solubility of cellulose, but has no positive effect on the yield of LA. The results illustrated that cellulose, as a renewable material, can be used to produce a high value-added chemical with the acid-catalyzed conversion under pressure reaction process in a biphasic solvent system.

## Data Availability Statement

The original contributions presented in the study are included in the article/Supplementary Material, further inquiries can be directed to the corresponding author/s.

## Author Contributions

All authors listed have made a substantial, direct and intellectual contribution to the work, and approved it for publication.

## Conflict of Interest

The authors declare that the research was conducted in the absence of any commercial or financial relationships that could be construed as a potential conflict of interest.
