# Supplementary material for: Acid-Catalyzed Conversion of Cellulose Into Levulinic Acid With Biphasic Solvent System
Source: Front Plant Sci. 2021 Mar 17;12:630807. doi: 10.3389/fpls.2021.630807 (PMC8010141; doi:10.3389/fpls.2021.630807)
Supplement: Supplementary file 1 [file Table_1.DOCX]

**Supporting Information**

**Table S1** Products distribution of different Fe_2_(SO_4_)_3_ dosage on cellulose conversion

| **Entry** | **Cellulose/mg** | **Catalyst/mg** | **Glucose/%** | **Fructose/%** | 1. **HMF/%** | **LA/%** | **Conversion/%** |
| --- | --- | --- | --- | --- | --- | --- | --- |
| 1 | 486 | 399 | 4.39 | 0.97 | 1.59 | 6.12 | 42.02 |
| 2 | 240 | 240 | 4.54 | 0.79 | 2.66 | 2.94 | 35.5 |
| 3 | 486 | 399 | 4.71 | 0.77 | 1.78 | 8.29 | 50.51 |
| 4 | 240 | 171 | 4.16 | 0.77 | 2.36 | 6.04 | 46.79 |
| 5 | 240 | 160 | 3.53 | 0.77 | 3.16 | 4.61 | 41.13 |
| 6 | 240 | 120 | 2.20 | 0.83 | 3.80 | 4.55 | 37.46 |

Reaction condition: H_2_O 12 mL, 180 ℃, 1: 120 min; 2-6: 180 min.

**Table S2** Products distribution of different Al_2_(SO_4_)_3_ dosage on cellulose conversion

| **Entry** | **Cellulose/mg** | **Catalyst/mg** | **Glucose/%** | **Fructose/%** | **5-HMF/%** | **LA/%** | **Conversion/%** |
| --- | --- | --- | --- | --- | --- | --- | --- |
| 1 | 486 | 666 | 0.06 | 0.01 | 0 | 19.66 | 69.44 |
| 2 | 486 | 666 | 0 | 0 | 0 | 20.33 | 70.49 |
| 3 | 240 | 240 | 0.49 | 0 | 0 | 21.24 | 72.4 |
| 4 | 240 | 171 | 0.64 | 0.02 | 0.16 | 23.35 | 71.88 |
| 5 | 240 | 160 | 0.87 | 0 | 0 | 22.21 | 75.83 |
| 6 | 240 | 120 | 1.88 | 0 | 1.78 | 21.56 | 73.54 |

Reaction condition: H_2_O 12 mL, 180 ℃, 1: 120 min; 2-6: 180 min.

**Table S3** Products distribution of different Cr_2_(SO_4_)_3_ dosage on cellulose conversion

| **Entry** | **Cellulose/mg** | **Catalyst/mg** | **Glucose/%** | **Fructose/%** | **5-HMF/%** | **LA/%** | **Conversion/%** |
| --- | --- | --- | --- | --- | --- | --- | --- |
| 1 | 486 | 500 | 0.88 | 0.41 | 1.55 | 7.02 | 23.31 |
| 2 | 486 | 500 | 1.42 | 0.66 | 1.55 | 8.87 | 34.2 |
| 3 | 240 | 240 | 1.34 | 0.69 | 1.93 | 8.19 | 26.6 |
| 4 | 240 | 171 | 2.34 | 0.98 | 2.58 | 6.69 | 25.63 |
| 5 | 240 | 160 | 1.97 | 0.73 | 2.64 | 6.51 | 32.71 |
| 6 | 240 | 120 | 2.22 | 0.66 | 3.16 | 4.93 | 28.96 |

Reaction condition: H_2_O 12 mL, 180 ℃, 1: 120 min; 2-6: 180 min.

**Table S4** Effect of different catalysts on cellulose conversion and LA yield

| **Entry** | **Catalyst** | **LA/%** | **Conversion/%** | **Selectivity of LA/%** |
| --- | --- | --- | --- | --- |
| 1 | AlCl_3_ | 25.16 | 83.27 | 30.21 |
| 2 | CrCl_3_ | 18.91 | 86.87 | 21.77 |
| 3 | FeCl_3_ | 31.78 | 84.53 | 37.60 |
| 4 | Al_2_(SO_4_)_3_ | 23.35 | 71.88 | 32.48 |
| 5 | Cr_2_(SO_4_)_3_ | 8.87 | 26.6 | 33.35 |
| 6 | Fe_2_(SO_4_)_3_ | 8.29 | 50.51 | 16.41 |
| 7 | HZSM-5 | 0.21 | 27.7 | 0.76 |

Reaction condition: cellulose 100 mg, catalyst 100 mg, H_2_O 12 mL, 180 ℃, 180 min.

Metal salt catalysts are less corrosive than mineral acid and some salts can provide both Lewis acid and Brønsted acid sites.^1^ Nevertheless, surface area and pore volume of the catalysts were less influential compared to Brønsted acidity and the quantity of the acid sites in this situation.^2^ As listed in **Table S4**, FeCl_3_ has the highest catalytic activity among all three chlorine salts studied in this work. The yield of LA is 31.78% and the conversion of cellulose is 84.53%. This result may be attributed to that FeCl_3_ catalyst can provide more active acid site than other chlorine salt catalysts, which would promote the cleavage of the glycosidic bond and release glucose.^1^ Among three sulfate salt catalysts, Al_2_(SO_4_)_3_ gave the highest yield of the target product LA (23.35%) with 71.88% conversion of cellulose. It has been reported that, in aqueous solution, Al_2_(SO_4_)_3_ could exhibit appropriate Brønsted acid/Lewis acid ratio to control the product selectivity, which functions both as the Lewis acid responsible for the isomerization of glucose into fructose and the Brønsted acid for the transform of fructose to LA with 5-HMF as intermediate compounds.^3^ Both chromium salts, especially Cr_2_(SO_4_)_3_ gave poor LA yields probably owing to the formation of the chromium–glucose chelate complex during the reaction restrained the conversion of intermediate glucose and resulted in a substantial drop in the LA yield.^4^ Our results show that chloride-based salts were more effective than its counterpart sulfate-salts, which is in agreement of known literature.^3,4^ Two commercially available solid acid catalysts, HZSM-5 and Amberlyst-15 were also used for the cellulose conversion to LA. A lower LA yield was obtained with H-ZSM-5 owing to its weak acidity in aqueous solution (0.21%).

**Table S5** Properties of solvents used in this work

| **Solvent** | **Chemical structure** | **Boiling point/℃** | **Melting point/℃** | **Density/g/mL** | **Water solubility/g/100 g** | **Flash point/℃** | **Polarity** |
| --- | --- | --- | --- | --- | --- | --- | --- |
| THF |  | 65 | -108.4 | 0.8833 | 30 | -14 | 0.207 |
| GVL |  | 207 | -31 | 1.05 | miscible | 96 | - |
| DMSO |  | 189 | 18.4 | 1.092 | 25.3 | 95 | 0.444 |
| 1,4-Dioxane |  | 101 | 11 | 1.033 | miscible | 12 | 0.164 |
| sulfolane |  | 285 | 28.45 | 1.261 | miscible | 166 | - |

**Table S6** Products distribution of different substrates

| **Substrate** | **Glucose/%** | **Fructose/%** | **5-HMF/%** | **LA/%** | **FA/%** | **Conversion/%** |
| --- | --- | --- | --- | --- | --- | --- |
| Glucose^a^ | 1.58 | 2.90 | 0 | 31.88 | 14.72 | 98.1 |
| Fructose^b^ | 0.42 | 0.63 | 0 | 38.92 | 13.76 | 98.2 |
| 5-HMF^c^ | 0 | 0 | 0 | 52.93 | 18.86 | 98.2 |

Reaction condition: a: glucose 240 mg, Amberlyst-15 500 mg, 160 ℃, 180 min, H_2_O 6 mL, GVL 6 mL; b: fructose 240 mg, Amberlyst-15 500 mg, 160 ℃, 180 min, H_2_O 6 mL, GVL 6 mL; c: 5-HMF 240 mg, Amberlyst-15 500 mg, 140 ℃, 180 min, H_2_O 6 mL, GVL 6 mL.

**Table S7** Effect of different biphasic solvent systems on products distribution

| **Solvent** | **Temp./℃** | **Glucose/%** | **Fructose/%** | **5-HMF/%** | **LA/%** | **Conversion/%** |
| --- | --- | --- | --- | --- | --- | --- |
| H_2_O | 180 | 0.04 | 0.004 | 0.70 | 29.91 | 71.29 |
| GVL/H_2_O | 180 | 0 | 0 | 0 | 36.90 | 93.83 |
| GVL/H_2_O | 200 | 0.18 | 0 | 0 | 50.40 | 83.54 |
| THF/H_2_O | 180 | 0 | 0 | 0 | 47.73 | 94.25 |
| THF/H_2_O | 200 | 0 | 0.36 | 0 | 34.80 | 87.16 |
| DIO/H_2_O | 180 | 0 | 0.09 | 0 | 32.18 | 81.04 |
| Sulfolane/H_2_O | 180 | 0 | 0 | 0 | 22.58 | 83.71 |
| DMSO/H_2_O | 180 | 0.84 | 1.46 | 0.79 | 5.69 | 42.33 |

Reaction condition: cellulose 100 mg, Amberlyst-15 300 mg, 180 min, solvent 12 mL, ratio of biphasic solvents: 1:1.

**Table S8** Effect of temperature on products distribution

| **Temp./℃** | **Glucose/%** | **Fructose/%** | **5-HMF/%** | **LA/%** | **Conversion/%** |
| --- | --- | --- | --- | --- | --- |
| 160 | 28.32 | 2.00 | 0 | 8.94 | 69.08 |
| 180 | 0 | 0 | 0 | 36.90 | 72.83 |
| 200 | 0.18 | 0 | 0 | 50.40 | 83.54 |
| 220 | 0.13 | 0.38 | 0 | 49.43 | 100 |
| 240 | 0 | 0 | 0 | 46.31 | 100 |

Reaction condition: cellulose 100 mg, Amberlyst-15 300 mg, H_2_O 6 mL, GVL 6 mL, 180 min.

**Table S9** Effect of time on products distribution

| **Time/min** | **Glucose/%** | **Fructose/%** | **5-HMF/%** | **LA/%** | **Conversion/%** |
| --- | --- | --- | --- | --- | --- |
| 120 | 0.21 | 0.57 | 0 | 37.56 | 72.04 |
| 150 | 0 | 0.98 | 0 | 46.51 | 78.23 |
| 180 | 0.18 | 0 | 0 | 50.40 | 83.54 |
| 210 | 0 | 0.82 | 0 | 47.00 | 93.98 |
| 240 | 0.22 | 0.37 | 0 | 39.48 | 99.01 |

Reaction condition: cellulose 100 mg, Amberlyst-15 300 mg, H_2_O 6 mL, GVL 6 mL, 200 ℃.

**Table S10** Effect of different biphasic solvent systems on products distribution

| **Solvent** | **N_2_/MPa** | **Glucose/%** | | **Fructose/%** | **5-HMF/%** | **LA/%** | **Conversion/%** |
| --- | --- | --- | --- | --- | --- | --- | --- |
| GVL/H_2_O | 0 | 0.18 | 0 | | 0 | 50.40 | 83.54 |
| GVL/H_2_O | 4 | 0 | 0.73 | | 0 | 54.21 | 97.17 |
| THF/H_2_O | 0 | 0 | 0 | | 0 | 47.73 | 94.25 |
| THF/H_2_O | 4 | 0.44 | 0.33 | | 0 | 54.49 | 96.58 |
| DIO/H_2_O | 0 | 0 | 0.09 | | 0 | 31.47 | 81.04 |
| DIO/H_2_O | 4 | 0 | 1.11 | | 0 | 32.06 | 85.84 |
| Sulfolane/H_2_O | 0 | 0 | 0 | | 0 | 22.58 | 83.71 |
| Sulfolane/H_2_O | 4 | 0 | 0.40 | | 0 | 23.85 | 88.91 |
| DMSO/H_2_O | 0 | 0.84 | 1.46 | | 0.79 | 5.69 | 42.33 |
| DMSO/H_2_O | 4 | 2.58 | 0.70 | | 0 | 15.79 | 60.63 |

Reaction condition: cellulose 100 mg, Amberlyst-15 300 mg, 180 min, 200 ℃, solvent 12 mL, ratio of biphasic solvents: 1:1.

**Table S11** Effect of catalyst loading on products distribution

| **ratio** | **Glucose/%** | **Fructose/%** | **5-HMF/%** | **LA/%** | **Conversion/%** |
| --- | --- | --- | --- | --- | --- |
| 1:0.5 | 0 | 0.79 | 0 | 32.79 | 97.98 |
| 1:1 | 0 | 0.86 | 0 | 58.45 | 98.29 |
| 1:2 | 0 | 0.86 | 0 | 59.24 | 99.7 |
| 1:3 | 0 | 0.73 | 0 | 54.21 | 97.17 |

Reaction condition: cellulose: Amberlyst-15, H_2_O 6 mL, GVL 6 mL, 180 min, 200 ℃, 4MPa N_2_.

**Fig. S1** Effect of N_2_ pressure on cellulose conversion

Reaction condition: cellulose 100 mg, Amberlyst-15 300 mg, H_2_O 6 mL, GVL 6 mL, 180 min, 200 ℃.

**Fig. S2** HPLC spectra of reaction solution

Reaction condition: cellulose 3 wt%, Amberlyst-15 6 wt%, H_2_O 6 mL, GVL 6 mL, 200 ℃, 180 min, 4 MPa N_2_.

**Fig. S3** Effect of different dosage of NaCl on cellulose conversion

Reaction condition: cellulose 3 wt%, Amberlyst-15 6 wt%, 200 ℃, 180 min, H_2_O 6 mL, GVL 6 mL, 4 MPa N_2_.

**Fig. S4** Effect of reuse of Amberlyst-15 on cellulose hydrolysis

Reaction condition: cellulose 3 wt%, Amberlyst-15 6 wt%, 200 ℃, 180 min, H_2_O 6 mL, GVL 6 mL, 4 MPa N_2_.

**Notes and references**

1. Huang, Y.-B., T. Yang, M.-C. Zhou, H. Pan., & Y. Fu. (2016). Microwave-assisted alcoholysis of furfural alcohol into alkyl levulinates catalyzed by metal salts. Green Chemistry, 18(6), 1516-1523.

2. Chen, S. S., Yu, I. K. M., Tsang, D. C. W., Yip, A. C. K., Khan, E., Wang, L., Ok, Y. S., & Poon, C. S. (2017). Valorization of cellulosic food waste into levulinic acid catalyzed by heterogeneous Brønsted acids: Temperature and solvent effects. Chemical Engineering Journal, 327, 328–335.

3. Huang, Y. B., Yang, T., Luo, Y. J., Liu, A. F., Zhou, Y. H., Pan, H., & Wang, F. (2018). Simple and efficient conversion of cellulose to γ-valerolactone through an integrated alcoholysis/transfer hydrogenation system using Ru and aluminium sulfate catalysts. Catalysis Science and Technology, 8(23), 6252–6262.

4. Nemoto, K., K.-i. Tominaga., & K. Sato. (2015). Facile and Efficient Transformation of Lignocellulose into Levulinic Acid Using an AlCl_3_•6H_2_O/H_3_PO_4_ Hybrid Acid Catalyst. Bulletin of the Chemical Society of Japan, 88(12), 1752-1754.
